# Changes in the Viscoelastic Properties of the *Vastus Lateralis* Muscle With Fatigue

**DOI:** 10.3389/fphys.2020.00307

**Published:** 2020-04-24

**Authors:** Emeric Chalchat, Jean-Luc Gennisson, Luis Peñailillo, Myriam Oger, Alexandra Malgoyre, Keyne Charlot, Cyprien Bourrilhon, Julien Siracusa, Sebastian Garcia-Vicencio

**Affiliations:** ^1^Unité de Physiologie de l’Exercice et des Activités en Conditions Extrêmes, Département Environnements Opérationnels, Institut de Recherche Biomédicale des Armées, Brétigny-sur-Orge, France; ^2^BIOMAPS, Laboratoire d’Imagerie Biomédicale Multi-Modale, CEA, Université Paris-Saclay, CNRS UMR 9011, INSERM UMR 1281, Orsay, France; ^3^Exercise Science Laboratory, School of Kinesiology, Faculty of Medicine, Finis Terrae University, Santiago, Chile; ^4^Unité Imagerie, Département des Plateformes et Recherche Technologique, Institut de Recherche Biomédicale des Armées, Brétigny-sur-Orge, France; ^5^LBEPS, Univ Evry, IRBA, Université Paris Saclay, Evry, France

**Keywords:** stiffness, viscosity, exercise, shear-wave spectroscopy, shear-wave elastography, isometric contractions, muscle compliance

## Abstract

We investigated the *in vivo* effects of voluntary fatiguing isometric contractions of the knee extensor muscles on the viscoelastic properties of the *vastus lateralis* (VL). Twelve young males (29.0 ± 4.5 years) performed an intermittent voluntary fatigue protocol consisting of 6 sets × 10 repetitions of 5-s voluntary maximal isometric contractions with 5-s passive recovery periods between repetitions. Voluntary and evoked torque were assessed before, immediately after, and 20 min after exercise. The shear modulus (μ) of the VL muscle was estimated at rest and during a ramped isometric contraction using a conventional elastography technique. An index of active muscle stiffness was then calculated (slope from the relationship between shear modulus and absolute torque). Resting muscle viscosity (η) was quantified using a shear-wave spectroscopy sequence to measure the shear-wave dispersion. Voluntary and evoked torque decreased by ∼37% (*P* < 0.01) immediately after exercise. The resting VL μ was lower at the end of the fatigue protocol (−57.9 ± 5.4%, *P* < 0.001), whereas the resting VL η increased (179.0 ± 123%, *P* < 0.01). The active muscle stiffness index also decreased with fatigue (*P* < 0.05). By 20 min post-fatigue, there were no significant differences from the pre-exercise values for VL η and the active muscle stiffness index, contrary to the resting VL μ. We show that the VL μ is greatly reduced and η greatly enhanced by fatigue, reflecting a more compliant and viscous muscle. The quantification of both shear μ and η moduli *in vivo* may contribute to a better understanding of the mechanical behavior of muscles during fatigue in sports medicine, as well as in clinical situations.

## Introduction

*In vivo* characterization of the viscoelastic mechanical properties of skeletal muscle [e.g., stiffness or shear modulus (μ) and shear viscosity (η)] has long been a great interest (Gennisson et al., 2010; Lacourpaille et al., 2012, 2014; Andonian et al., 2016; Davis et al., 2018; Rasool et al., 2018). Recent evidence suggests that the assessment of subtle modifications of muscle stiffness may be helpful in identifying the early development of muscle fatigue (Andonian et al., 2016; Siracusa et al., 2019), damage (Lacourpaille et al., 2014), or disease (Rasool et al., 2018). We recently demonstrated (Siracusa et al., 2019) that the resting *vastus lateralis* μ (VL μ), assessed by shear-wave elastography (SWE), was reduced (−34.7 ± 6.7%) after a series of voluntary isometric contractions of the knee extensor (KE) muscles. Moreover, such reduced muscle stiffness was accompanied by a decrease in the voluntary torque and modification of the mechanical properties of a single twitch (e.g., reduced peak torque, longer electromechanical delay and contraction time, and reduced rate of force development) of the KE after exercise. These results suggest that measured modifications in resting muscle stiffness can be used to complement traditional neuromuscular measurements to estimate changes in the mechanical properties of skeletal muscle with fatigue. Aside from such studies under resting conditions, the study of active muscle stiffness may provide valuable information about patterns of muscle fatigue during exercise. Identifying patterns of muscle fatigue related to changes in muscle stiffness could help diagnose muscle states (e.g., neuromuscular disorders) or provide insights for muscle rehabilitation in athletes. However, conflicting results have been reported. Indeed, decreases in active stiffness with fatigue have been confirmed after dynamic and intermittent submaximal isometric contractions (Vigreux et al., 1980; Morel et al., 2019), but not after sustained isometric protocols (Bouillard et al., 2012a; Akagi et al., 2019). Akagi et al. (2019) showed an increase in resting VL μ (21.7 ± 32.4%) after a low-intensity prolonged fatiguing task of the KE muscles. Akagi et al. (2017) suggested that resting muscle stiffness could be higher after prolonged fatiguing task due to muscle failure to fully relax and cramps. Moreover, it is not known whether the changes in viscoelastic properties of muscles contribute to restoration of force transmission capacities during the recovery period. The well-known task dependency of fatigue needs to be considered to better understand discrepancies concerning changes in muscle stiffness immediately after fatiguing exercise and during the recovery period.

Studies on muscle viscoelasticity have mainly focused on characterizing elastic behavior, largely neglecting the viscous component. The concept of muscle η was first introduced by Hill (1922) and was defined as the resistance (frictional forces or energy dissipative elements) of fluid to flow when it is subjected to shear stress. It has been suggested that the viscous properties of muscle, such as that of the myoplasm, concentrations of inorganic phosphate, and weakly attached cross-bridges, collagen fibrils, titin, and other cytoskeletal proteins (Mauro and Adams, 1961; Moss and Halpern, 1977; Stackhouse et al., 2001), influence the capacity to develop maximal tension and relaxation (Meyer et al., 2011). It is plausible to assume that a viscous muscle would take much longer to adapt to a mechanical stress than a less viscous muscle. Furthermore, muscle η may be affected by muscle fatigue (Zhang and Rymer, 2001), which is important, because higher η may be related to reductions in muscle fiber relaxation and the detachment rate of cross-bridges, affecting muscle function. Changes in muscle η with fatigue may affect the natural capacity of muscles to absorb mechanical shock and prevent overly sudden changes in tension. However, the *in vivo* characterization of muscle η and its modification with fatigue has not been systematically investigated in humans, and current methods are limited.

The ultrasound SWE approach provides an opportunity to quantify the viscoelastic properties of muscle *in vivo* by assessing both the local shear modulus μ and viscosity η from the frequency-dependent changes of the shear-wave speed (i.e., dispersion) using rheological models (Catheline et al., 2004; Deffieux et al., 2009; Gennisson et al., 2010; Rasool et al., 2018). Shear-wave signal processing is known as shear-wave spectroscopy (SWS). Rheological models are based on the study of both elastic (spring) and viscous (dashpot) elements connected in various series and parallel configurations, assuming specific criteria, including linearity, homogeneity, and isotropy of the muscle tissue. However, this method has been rarely employed to investigate the specific viscoelastic properties of muscle in humans.

Therefore, we aimed to investigate the effects of repeated voluntary maximal isometric contractions of the KE muscles on resting and active shear modulus and viscosity of the VL muscle *in vivo* immediately after exercise and during recovery. We hypothesized that high-intensity exercise would induce a high level of peripheral fatigue affecting the viscoelastic properties (reduced stiffness and higher viscosity) of the VL as well as the KE force transmission capacity.

## Materials and Methods

### Participants

Twelve young males (age: 29.0 ± 4.5 years, body mass: 81.6 ± 7.1 kg, and height: 176.0 ± 8.0 cm) provided their written informed consent and volunteered to participate in the present study. Participants performed regular physical activity, such as strength training, running, and/or cross-training (between 6 and 15 h/w), with no recent history of muscular, joint, or bone disorders or of receiving any medication that could interfere with neuromuscular responses. All volunteers were fully informed of the experimental procedures, aims, and risks and gave their written informed consent before any testing was conducted. Each participant participated in an inclusion session, consisting of a complete medical examination, including the collection of anthropometric data and complete familiarization with the experimental procedures. This study was approved by the scientific leadership of the French Armed Forces Biomedical Research Institute and the local ethics committee (CPP Ile de France VI). All experiments were conducted in accordance with the Helsinki Declaration.

### Protocol

**FIGURE 1:**
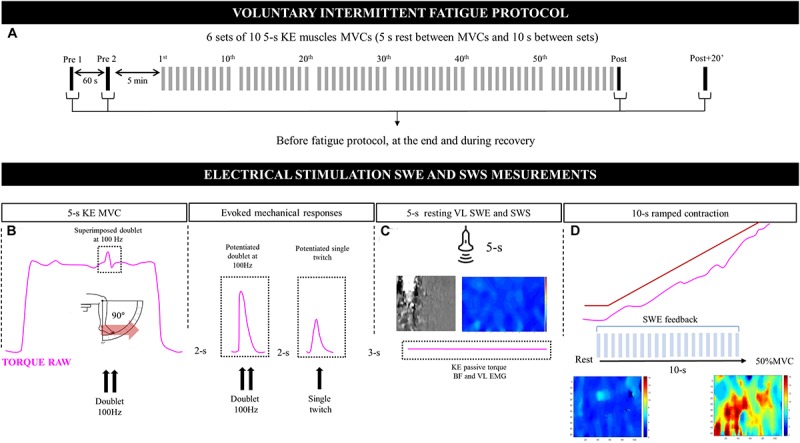
Design of the voluntary intermittent fatigue protocol (top; **A**), consisting of a series of voluntary force, electrical stimulation, and muscle viscoelasticity measurements performed before and after the fatigue protocol (bottom; **B**–**D**). KE, knee extensors; MVC, maximal voluntary contraction; SWE, shear-wave elastography; EMG, surface electromyography; VL, *vastus lateralis*; BF, *biceps femoris*; SWS, shear-wave spectroscopy.

Participants performed an intermittent voluntary fatigue protocol consisting of 6 sets × 10 repetitions of 5-s maximal voluntary isometric contractions (MVICs) of the KE muscles and 5-s passive recovery periods between repetitions with 10 s of rest between sets (Figure 1A). The number of contractions was chosen to generate a high level of voluntary strength loss and peripheral fatigue, as previously demonstrated (Siracusa et al., 2019). Participants were not informed of the criterion for the end of the task (60 MVCs) but had visual feedback of torque output during the exercise. They were also strongly encouraged by the researcher during the entire fatiguing task and testing. Peripheral fatigue and alterations in the contractile mechanical properties of muscles were determined by delivering double (Db100Hz) and single (Tw) electrical stimulations to the femoral nerve before, immediately after, and 20 min after the MVICs at rest (post + 20’; Figures 1A,B). The viscoelastic mechanical properties of the VL muscle (shear μ and η moduli) were evaluated after each electrical-stimulation series (Figure 1C). Then, one 10-s submaximal ramped isometric contraction (from rest to 50% MVC) of the KE muscle was performed (Figure 1D). VL muscle was chosen because previous studies (Alkner et al., 2000; Place et al., 2007) showed that this muscle is representative of the muscle quadriceps. Moreover, studies showing decreases in muscle stiffness by assessing aponeurosis or muscle tendon junction displacements or by SWE after repeated isometric contractions were made exclusively in the VL muscle (Kubo et al., 2001a; Morel et al., 2019; Siracusa et al., 2019), allowing comparisons with the present study.

### Measurements

#### Neuromuscular Outcomes

Participants were comfortably positioned on an adjustable chair with the hip joint flexed at 70° (90° = neutral “sitting” position). Before the voluntary fatigue protocol, two 5-s MVICs of the KE were performed, with 60-s passive recovery. The absolute MVIC peak torque was determined as the peak force reached during maximal efforts and was defined as the control “non-fatigued” value. All measurements were taken from the participant’s dominant leg (right leg for all participants), which was fixed at 90° (0° = knee fully extended). During each MVIC, participants were instructed to grip the seat to stabilize the pelvis. The double pulse (at 100 Hz) superimposition technique, based on the interpolated-twitch method (Merton, 1954), enabled us to estimate the maximal voluntary KE activation level. Square-wave pulses with a width of 1 ms at a maximal voltage of 400 V were delivered percutaneously to the femoral nerve using an electrical stimulator (Digitimer DS7A, Welwyn Garden City, United Kingdom) at supramaximal intensity. Intensity ranged from 55 to 99 mA, corresponding to 130% of the optimal intensity (i.e., the intensity at which maximal non-potentiated single twitches started to plateau). Intensities were determined from a progressive recruitment curve. Then, the following parameters were obtained from the potentiated single-twitch response: peak torque (Tw_*pot*_), electromechanical delay (EMD), contraction time (CT), half relaxation time (HRT), maximal rate of torque development (MRTD), and maximal rate of torque relaxation (MRTR). Passive KE torque was measured during each SWE measurement at a knee angle of 90° in order to ensure the relaxed state of the participant and to have an indication of the passive tension which could be interpreted as a global quadriceps muscle–tendon stiffness. The surface electromyography (EMG) signals (root mean square value of 300 ms) of the VL and biceps femoris (BF) muscles were recorded to evaluate any muscle activity generated during the resting SWE measurements. The bipolar silver chloride surface electrodes (Blue Sensor N-00-S, Ambu, Denmark) were taped lengthwise to the skin over the muscle belly, as recommended by SENIAM (Hermens et al., 2000), with an inter-electrode distance of 20 mm. Electrodes were placed at 2/3 on the line from the anterior spina iliaca superior to the lateral side of the patella for the VL muscle and at 50% on the line between the ischial tuberosity and the lateral epicondyle of the tibia for the BF muscle. The reference electrode was attached to the patella. Low impedance (Z < 5 kΩ) at the skin–electrode surface was obtained by shaving, gently abrading the skin with fine-grain sandpaper, and cleaning with alcohol. EMG signals were amplified (Dual Bio Amp ML 135, ADInstruments, Australia) with a bandwidth frequency ranging from 10 to 500 Hz (common mode rejection ratio > 85 dB, gain = 1,000) and simultaneously digitized together with the torque signals. The sampling frequency was 2 kHz. The temperature of the experimental room was controlled (20°C).

#### Shear-Wave Elastography Measurements

An ultrafast ultrasound scanner (Aixplorer version 12.2; Supersonic Imagine, Aix-en-Provence, France) coupled with a linear transducer array (SuperLinear 15-4; Supersonic Imagine, Aix-en-Provence, France) was used in the SWE mode (musculoskeletal preset, penetration, no persistence), as previously proposed (Bercoff et al., 2004). SWE and SWS measurements were carefully standardized. The B-mode ultrasound was first set to determine the optimal transducer location and maximize the alignment between the transducer and the direction of the muscle fascicles. Transducer alignment was considered to be correct when VL muscle fascicles and aponeurosis could be delineated across the image without interruption. The transducer was fixed using a dynamic probe fixation device (with 360° adjustments, USONO, Eindhoven, Netherlands) placed over the skin, which was coated with a water-soluble transmission gel (Aquasonic, Parker Laboratory, Fairfield, NJ, United States) to ensure acoustic coupling. Then, a fixed-size square region of interest (ROI; ∼1.5 cm^2^), i.e., a region in which shear-wave propagation was analyzed within the muscle, was placed in the middle of the B-mode image below the superficial aponeurosis within the VL. A two-dimensional (2D) real-time stiffness (μ shear modulus) color map was obtained with a frame rate of 1.5–2 Hz and a spatial resolution of 1 mm^2^ × 1 mm^2^, as described above (Figure 1C). Values were averaged over the largest ROI (a ∼36 × 114 matrix), and the average of the obtained consecutive matrix was used for subsequent analyses for resting and active measurements.

Two types of sequences were used to estimate the shear modulus μ and shear viscosity η of the medium: (i) the conventional SWE sequence to generate a full image of the local shear-wave velocity (SWV) group at rest and during ramped contractions for the shear modulus μ (Gennisson et al., 2010) and (ii) the SWS sequence to measure the shear-wave dispersion to estimate the muscle shear viscosity η (Deffieux et al., 2009). The last sequence was only available for the static condition due to technological limitations. Thus, muscle shear viscosity η was assessed only at rest.

#### Shear-Wave Elastography Sequence

**FIGURE 2:**
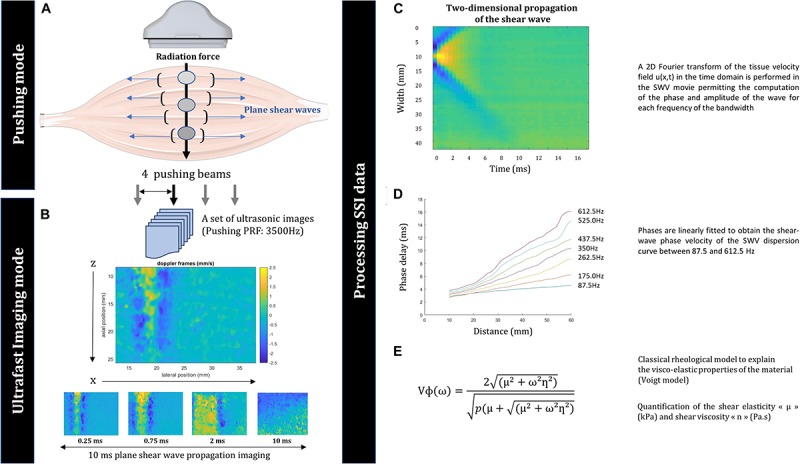
Schema of the imaging setup with the ultrasound transducer **(A)**. Ultrafast imaging mode: the propagation of the planar shear waves of the first pushing beam is shown over 10 ms **(B)**. Processing of the supersonic imaging data: velocity field of the shear-wave velocity along the fibers **(C)**, relationship between the phase delay (ms) and the distance (mm) **(D)**, and rheological model used to explain the viscoelastic properties of muscle **(E)**.

The SWE technique consists of a transient and remote palpation generated by the radiation force induced by a focused ultrasonic beam (Figure 2A). Each ultrasonic beam generates a remote vibration at different depths that results in the propagation of a transient shear-wave (Bercoff et al., 2004). After generation of the shear-wave, an ultrafast ultrasound imaging sequence is performed to acquire successive raw radio-frequency data at a very high frame rate (up to 20,000 frames/s), contrary to conventional ultrasonography (typically 50 frames/s; Figure 2B). A map of the local shear group velocity was estimated for each of the four pushing lines in each pixel of the resulting image based on the displacement moves using a time-of-flight algorithm. The shear-wave propagation velocity, typically a few meters per second in soft tissue, correlates directly with muscle μ if the medium is assumed to be purely elastic, homogeneous, and locally isotropic in the imaging plane, which is well-accepted in muscle elastography studies (Bercoff et al., 2004; Catheline et al., 2004). The μ was obtained as follows:

$$\mu =\rho .V\,s^{2}$$

where ρ is the muscle density (1,000 kg/m^3^) and *V*_*s*_ is the shear-wave speed (in m/s). This equation implicitly neglects viscous effects (assuming the medium to be non-dispersive).

#### Resting and Active Muscle Stiffness Measurements

A 5-s SWE sequence was performed at rest for the calculation of the resting VL μ prior to the active measurements, for which the 2D real-time color map was maximally homogeneous. Then, the μ was calculated under the same experimental conditions throughout an incremental isometric task, consisting of a 10-s smooth linear force ramp from 0 to 50% MVIC (adjusted at each time point after exercise: post and post + 20’). A 5V TTL signal was triggered at the generation of each SWE image using a PowerLab system (16/30-ML880/P, ADInstruments, Bella Vista, Australia) connected to the ultrasound device to synchronize the SWE measurements with the torque values. During the ramped contractions, the cutoff was set at 50% MVIC, corresponding to the upper limit, after which the elastography measurement reaches saturation for up to 25% of the ROI (saturation = 16.329 m/s or 800 kPa), as observed in a previous study (Siracusa et al., 2019). An active muscle stiffness index was then calculated from a linear regression between the mean VL μ values and KE absolute torque (slope), which is independent of the level of force. All SWE measurements were transferred to a workstation and analyzed using a MATLAB (MathWorks, Natick, MA, United States) script developed in our laboratory.

#### Shear-Wave Spectroscopy Sequence

The SWS sequence, dedicated to the measurement of shear-wave dispersion (phase velocity versus frequency), corresponds to the previously proposed sequence (Deffieux et al., 2009). After each pushing beam (× 4) induced by the ultrasonic probe, the acquired velocity field *u(x,t,z)* (Figure 2B) of the SSI acquisition was averaged along the depth *z*. Then, the 3D tissue velocity field *u(x,t,z)* was transformed into a 2D tissue velocity field *u(x,t)*, describing the propagation of the plane wave in the square box along the lateral distance *x* (Figure 2C). A Fourier transform in the time domain was performed on the velocity field, allowing computation of the phase of the shear wave for each frequency of the bandwidth (between 87.5 and 612.5 Hz). Phases were linearly fitted as a function of the lateral distance to obtain the SWV dispersion curve (Figure 2D). These velocity measurements were then related to the viscoelastic properties of muscle using a viscoelastic model (Voigt; Figure 2E). The Voigt model links the applied stress σ to the resulting strain ε with the well-known equation:

σ=μ⁢ε+η⁢∂⁡ε

Under the assumption of planar shear-wave propagation, this governing equation leads to the expression of the phase velocity as (Chen et al., 2004):

$$V\,\phi \,\left(\omega \right)=\frac{\sqrt{2\,\left(\mu^{2}+\omega^{2}\,\eta^{2}\right)}}{\rho (\mu +\sqrt{\left(\mu^{2}+\omega^{2}\,\eta^{2}\right)}}$$

A non-linear optimization technique, such as the classical Nelder–Mead technique (Nelder and Mead, 1965), makes it possible to estimate both μ and η from the measurement of the dispersion law V_η_(ω). We used this approach to calculate a global index of the muscle η under passive conditions. One SWS sequence was triggered (Figure 1B), solely at rest, before the SWE measurements to calculate the shear η. The slopes of the linear regressions obtained between the phase velocity and frequencies were used to calculate the shear-wave dispersion index.

### Statistical Analysis

A sample size of 3 (12 subjects included) was deemed sufficient to achieve high statistical power. This was based on the MVC effect size calculated from our previous study (*n* = 15; Siracusa et al., 2019) using the sample size calculator G^∗^Power 3.1.9.2. The sample size calculation was performed *a priori* with an F test for ANOVA: repeated measures, within factors and an effect size of 2.074, an α error probability of 0.05, a power of 0.95, one group and three measurements, a correlation among repeated measures of 0.5, and a non-sphericity correction e of 1.

The data were screened for normality of the distribution and homogeneity of variances using the Shapiro–Wilk normality and Levene tests, respectively. Differences in absolute values of the passive μ, η, slopes, and neuromuscular outcomes were compared by one-way ANOVA (effect:time). Coefficients of determination (R^2^) were calculated for each linear fitting. Results with a *P*-value < 0.05 were considered to be significant. For VL μ and η, interclass correlation coefficient (ICC; 1,1 case), standard error of the mean (SEM), and coefficient of variation (CV) were also evaluated. Statistical procedures were performed using Statistica 8.0 software (Statsoft, Inc., United States). The results are presented as the means ± SD for the table, text, and figures.

## Results

### Repeatability of Measurements

For VL μ, ICC, SEM, and CV values varied from 0.93 to 0.95, 0.4 to 0.7 kPa, and from 15.3 to 21% for the VL muscle. For VL η, ICC, SEM, and CV values varied from 0.94 to 0.95, 0.5 to 0.6 Pa × s, and from 20.6 to 46.9% for the VL muscle.

### Neuromuscular Outcomes

One-way ANOVA showed a significant absolute (relative to the “non-fatigued” control value) time-dependent effect on voluntary and evoked torque (*P* < 0.001). MVC, Db100Hz, and Tw_*pot*_ decreased by 36.2 ± 8.7% (*P* < 0.01), 34.7 ± 8.8% (*P* < 0.001), and 42.5 ± 11.5% (*P* < 0.001), respectively, by the end of the exercise phase of the protocol (*P* < 0.001). Mechanical properties of the Tw_*pot*_ (CT, MRTD, HRT, and MRTR) were also altered by fatigue (*P* < 0.05). Only MVC torque and HRT returned to baseline at post + 20’. The voluntary KE activation level was not significantly different at the end of the fatigue protocol or during recovery. Passive KE torque and resting EMG activity of the VL and BF muscles assessed during the SWE measurements remained unchanged during the fatigue protocol and recovery period. All absolute values are presented in Table 1.

**TABLE 1:** Absolute values for both neuromuscular and viscoelastic outcomes before and after exercise.

	**Pre**	**Post**		**Post + 20’**	
KE MVC (Nm)	336.751.5	213.637.2	***	310.046.9	
**Nervous factors**					
KE VAL (%)	88.24.5	80.913.7		87.54.5	
**Muscular factors**					
KE Db100Hz_pot (Nm)	118.013.8	77.114.4	***	97.219.1	*
KE Tw_pot (Nm)	80.911.7	46.511.6	***	51.614.3	***
EMD (ms)	35.410.6	43.613.2		40.312.2	
CT (ms)	39.68.3	46.410.4	*	42.69.7	*
MRTD (Nm.s)	1.80.4	1.00.3	***	1.10.3	***
HRT (ms)	97.240.7	108.044.1	*	79.826.1	
MRTR (Nm.s)	6.51.6	3.41.3	***	2.81.1	***
Shear viscosity (Pa.s)	6.54.3	14.96.6	**	8.94.0	
Shear elastic modulus (kPa)	8.81.4	5.20.5	***	8.21.2	***
**Resting neuromuscular outcomes**					
KE Passive Torque (Nm)	0.37980.1641	0.79370.8977		0.45190.2442	
VL resting RMS (mV)	0.00430.0085	0.00340.0058		0.00300.0045	
BF resting RMS (mV)	0.00150.0002	0.00150.0002		0.00160.0003	

Mean ± SD. KE, knee extensors; MVC, maximal voluntary contraction; VAL, voluntary activation level; Db100Hz_pot, potentiated double pulse evoked at 100 Hz; Tw_pot, potentiated single pulse; EMD, electromechanical delay; CT, contraction time; MRTD, maximal rate of torque development; HRT, half relaxation time; MRTR, maximal rate of torque relaxation; VL, *vastus lateralis*; BF, *biceps femoris*; RMS, root mean square.*P < 0.05, **P < 0.01, and ***P < 0.001, significantly different from pre-exercise.

### The Relationship Between Shear-Wave Velocity and Frequency

**FIGURE 3:**
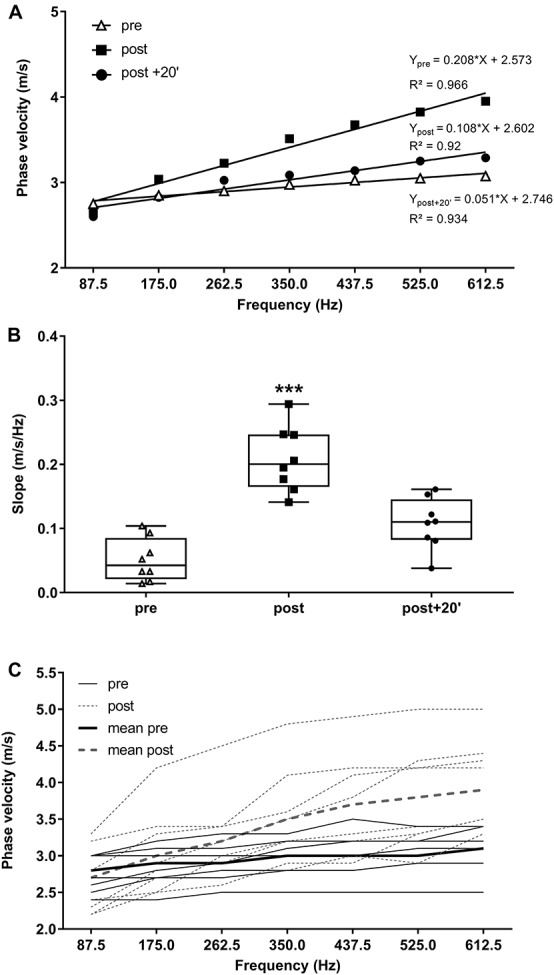
Phase velocity (m/s) along the fibers as a function of frequency (Hz) at pre, post, and post + 20’. Regressions equations and correlation coefficients are presented for each relationship **(A)**. Mean of the slopes for each time point and individual values. Mean ± SD (**B**,**C,** respectively). ^∗∗∗^*P* < 0.001, significant differences from pre-exercise (pre) values.

We quantified the local shear viscosity η from the frequency-dependence changes of the SWV (dispersion) using Voigt’s model. ANOVA showed a significant time effect for the phase velocity versus frequency relationship (*P* < 0.001; Figure 3). With fatigue, the mean of the slope was 682.4 ± 646.6% higher immediately after exercise and 360.1 ± 383.8% higher at post + 20’ than the pre-exercise values.

### Resting Viscosity

ANOVA showed a significant time-dependent effect for absolute resting VL η (*P* < 0.05; Figure 5A). Resting VL η values increased by the end of the fatigue protocol (179.0 ± 123.0%, *P* < 0.01) but returned to baseline by post + 20’. Individual values for the resting η are presented in Figure 5C.

### Resting Vastus Lateralis μ and Active Muscle Stiffness Index

**FIGURE 4:**
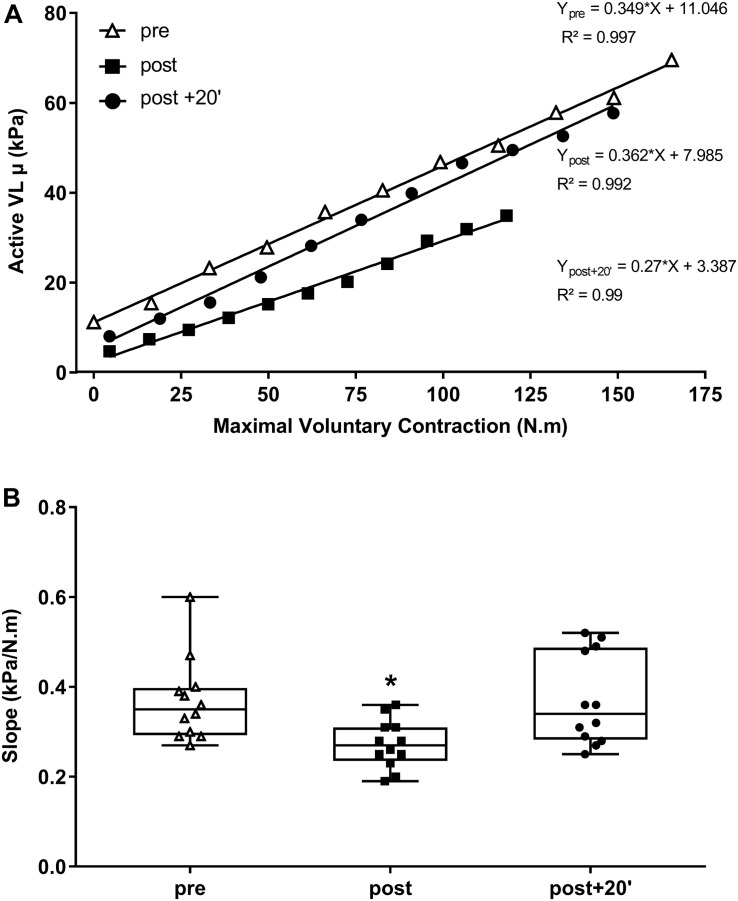
Active *vastus lateralis* (VL) muscle shear modulus (μ) and absolute torque relationship of the knee extensor muscles evaluated before and after exercise and during the recovery period (post + 20’). Regression equations and correlation coefficients are presented for each relationship **(A)**. The mean of the slope for each time point and individual values are given **(B)**. Mean ± SD **(B)**. *P < 0.05, significant differences from pre-exercise (pre) values.

**FIGURE 5:**
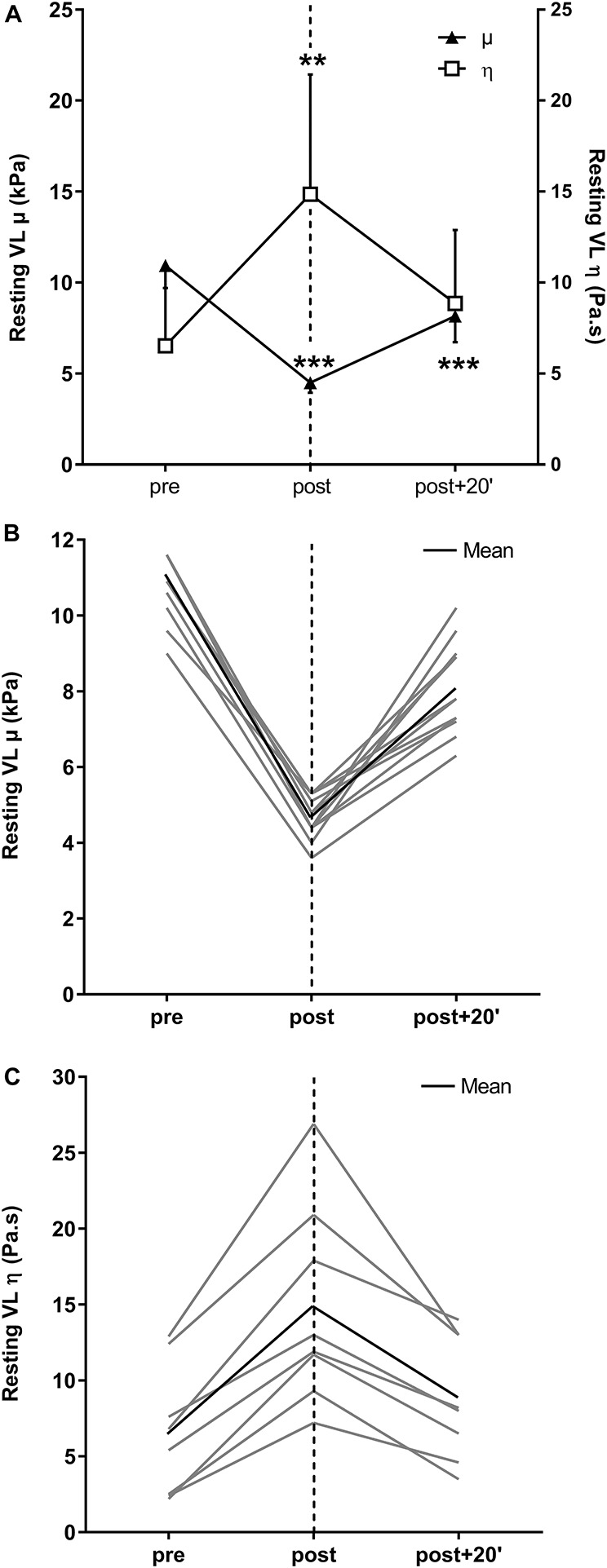
Shear elasticity (μ) and viscosity (η) of the *vastus lateralis* (VL) muscle extracted from the group velocity and Voigt’s model using the dispersion measurement **(A)**. Individual values for μ **(B)** and η **(C)**. Mean ± SD. **P < 0.01 and ***P < 0.001, significant differences from pre-exercise (pre) values.

ANOVA showed a significant time-dependent effect for the resting VL μ (*P* < 0.001; Figure 5A). The resting VL μ was significantly lower at the end of the exercise (−57.9 ± 5.4%, *P* < 0.001) and recovery periods (−27.0 ± 10.4%, *P* < 0.001). With fatigue, the active muscle stiffness index was 23.5 ± 17.5% lower immediately after exercise and 1.8 ± 23.4% lower at post + 20’ than the pre-exercise values (Figure 4).

## Discussion

The main results of this study were that muscle fatigue induces alterations of the viscoelastic properties of the VL (more compliant and viscous muscle). We observed ∼57% reduction of the resting VL μ and ∼23% lower active muscle stiffness index (slope) after intermittent maximal isometric KE contractions. Moreover, we found that muscle η increased by ∼150% with fatigue. The viscoelastic properties (η and active muscle stiffness index) progressively returned to baseline values by 20 min after exercise (at post + 20’), contrary to the VL μ and KE-evoked mechanical responses (Db100Hz and Tw_*pot*_), which remained reduced.

### Repeatability of Measurements

This study is the first to address the interest of η on a non-contracted muscle (VL) before and after a series of fatiguing isometric MVCs. Methodological factors such as slight probe motion, compression, variability of the measurement, and the ability of subjects to achieve a fully relaxed state were meticulously controlled to ensure good reliability of the measurements (Lacourpaille et al., 2012; Eby et al., 2013). There was little variability of the measurements before and after the fatigue protocol. The reproducibility values were obtained using five images (VL μ) and two sequences (VL η) at each point. For VL μ, ICC, SEM, and CV values varied from 0.93 to 0.95, 0.4 to 0.7 kPa, and from 15.3 to 21% for the VL muscle. We achieved this level of reproducibility by positioning a dynamic probe fixation system over the skin onto the VL muscle (Siracusa et al., 2019), avoiding any muscle deformation. For VL η, ICC, SEM, and CV values varied from 0.94 to 0.95, 0.5 to 0.6 Pa × s, and from 20.6 to 46.9% for the VL muscle. This level of reproducibility is slightly higher than μ possibly due to the anisotropy of muscle tissue. In addition, the VL and BF resting RMS and passive torque did not change during the SWE recordings (Table 1), verifying that the participants were in a relaxed state.

### Viscoelastic Properties of Muscles After High-Intensity Fatiguing Exercise

#### Muscle Stiffness

It is well-known that high-intensity exercise induces high peripheral fatigue (Lattier et al., 2004). Peripheral fatigue is generally characterized by altered E–C coupling and calcium kinetics (release and sensitivity), lower intrinsic force and fewer cross-bridges, increased concentrations of inorganic phosphate, etc. (Allen et al., 2008). Moreover, it is classically represented by a reduction in the magnitude of evoked responses (e.g., single twitch) and changes in their mechanical properties (e.g., higher contraction time, lower twitch relaxation time, and velocity) as observed in the present study.

Previous studies have also shown that repeated isometric fatiguing contractions reduce muscle–tendon stiffness, which decreases the force transmission capacity (Kubo et al., 2001a, b; Bojsen-Moller et al., 2003). However, the approach used in these previous studies (i.e., tracking of the muscle–tendon junction) may not be ideal as changes in stiffness mainly reflect modifications of the tendon properties rather than those of muscle. It has been largely demonstrated that SWE provides a reliable quantitative measurement of individual muscle–tissue stiffness (Lacourpaille et al., 2012; Brandenburg et al., 2015; Andonian et al., 2016). Moreover, there is a strong linear relationship between individual muscle force (passive and active) and muscle μ (Bouillard et al., 2011), providing an accurate index of individual muscle force, even when muscle fatigue occurs (Bouillard et al., 2012b).

It has been recently reported that VL μ is significantly reduced after maximal (−34.7 ± 6.7% at rest; Siracusa et al., 2019) and submaximal (−22 ± 7% during contractions; Morel et al., 2019) voluntary fatiguing isometric contractions of the KE muscles. Andonian et al. (2016) showed a decrease (∼−8%) in resting μ of the quadriceps muscle (VL, RF, and VM) after ultra-endurance running, which was still reduced more than 48 h (∼−5%) after exercise. These results are in accordance with those observed in the present study, showing that resting VL μ was significantly reduced immediately after (−57.9 ± 5.4%) and 20 min after exercise (post + 20’). However, our results are in contrast to a previous study (Akagi et al., 2019), which showed an increase in resting VL μ (21.7 ± 32.4%) after a low-intensity prolonged fatiguing task of the KE muscles. Akagi et al. (2017) suggested that resting muscle stiffness could be higher after prolonged fatiguing task due to muscle failure to fully relax and cramps. The well-known task dependency of fatigue needs to be considered to better understand discrepancies concerning changes in muscle stiffness and fatigue. The VL active muscle stiffness index (slope) was also reduced with fatigue (−27.0 ± 10.4% relative to baseline). These results are in accordance with those showing ∼12% reduction of active stiffness of the VL after sustained isometric (Morel et al., 2019), dynamic (Vigreux et al., 1980; Hunter and Kearney, 1983), and intermittent isometric (submaximal) contractions (Zhang and Rymer, 2001). There are several possible explanations for these phenomena. It is possible that muscle fatigue induced a decrease in muscle stiffness of the contractile elements due to the inability of sarcomere cross-bridges to generate as much net force as they produced before fatigue for the same level of external stretch. This was confirmed by Morel et al. (2019), who showed that the VL shear modulus was significantly reduced (∼16% lower) at a matched level of absolute force (40% MVIC; from Pre-) after submaximal sustained isometric contractions (∼15 min at 60% MVIC). However, the shear modulus was not assessed at a given absolute torque level after exercise in this study, limiting the conclusions.

Another possible mechanism allowing to explain the reduction in muscle stiffness with fatigue is the modification of the intramuscular temperature with repeated maximal effort, which may also induce a decrease in muscle stiffness. Indeed, a previous study suggested that the increased muscle temperature might contribute to 20–25% of the EMD elongation found during the fatiguing intermittent exercise (Zhou et al., 1998). As well as the force transmission capacities of muscles may depend on both mechanical and electrochemical processes (membrane excitability, muscle fiber conduction velocity), it has been suggested that any alteration on the electromechanical properties of the skeletal muscle is mainly associated with elastic rather than electrochemical processes (Ce et al., 2013, 2015). Second, the elongation of the connective structures, with fatigue could be attributable to an acute change in the arrangement of the collagen fibers (Stromberg and Wiederhielm, 1969) or alteration of the viscous properties of the intramuscular connective tissue as a result of increased muscle temperature (Strickler et al., 1990). Third, the ratio of force to stiffness cross-bridge may vary in the presence of fatigue induced by repeated contractions. Indeed, it has been suggested that stiffness at the sarcomere level is related to the myofilament mechanical properties themselves (Edman, 2009; Colombini et al., 2010). As such, several studies have demonstrated that the attached myosin heads include both the force-generating and the non-force-generating heads (Tsaturyan et al., 2011), with the latter contributing to the stiffness but not to force generation. Finally, the myosin power stroke does not only produce force in the axis of shortening but a radial component also exists (i.e., orthogonal to the long axis of the myofilaments; Williams et al., 2010). This radial component varies with the lattice spacing (i.e., the radial spacing between the contractile filaments), which is influenced by the osmotic pressure as a result of metabolic by-product accumulation during repeated contractions (Rapp et al., 1998). Further studies are needed to bring new insights into the mechanisms that may be involved in the decrease in active and passive muscle stiffness due to fatigue in both radial and longitudinal axes.

The results of the first part of our study confirm that repeated maximal fatiguing exercise induces a reduction in muscle stiffness (a decrease in both the resting and active μ), which could be responsible, at least in part, for the deficit in the capacity of force transmission (highlighted by the increased CT and reduced MRTD) with fatigue. Conversely, we found that there were no significant changes in KE passive torque, despite the reduction in VL muscle stiffness. It could be that muscular changes were too low to affect passive torque, especially at short or neutral muscle length (knee angle of ≤ 90°). Indeed, Lacourpaille et al. (2014) showed that changes in shear elastic modulus of biceps brachii was higher at long muscle length than short muscle length after 1 h of exercise. Moreover, it is possible that muscle fatigue may have induced a change in load sharing among the KE muscles, with a greater extent of fatigue in the VL muscle affecting its intrinsic force. For example, Bouillard et al. (2014) reported that the VL μ evaluated during a matched sustained submaximal isometric contraction of the KE muscles was lower after a fatiguing exercise, without changes in the other heads of the quadriceps, partially confirming this hypothesis.

#### Muscle Viscosity (η)

Studies on the viscoelasticity of muscle have mainly focused on characterizing elastic behavior, largely neglecting the viscous component. However, it is known that severe muscle fatigue may induce an increase in joint η (Zhang and Rymer, 2001). It is plausible to assume that a viscous muscle would take much longer to adapt to a mechanical stress than a less viscous muscle. A higher η after fatiguing exercise may be related to reduced relaxation of the muscle fibers and a lower cross-bridge detachment rate, affecting muscle function (Westerblad and Allen, 1993). This would affect the natural capacity of muscles to absorb mechanical shocks and prevent overly sudden changes in tension. Although the relationship between the level of muscle viscosity and the degree of protection is still unknown, we could reasonably suggest that any modification of the steady state of the muscle tissue could affect the optimal functioning of the muscle–tendon unit that may promote injuries.

The estimation of muscle shear viscosity η is much more complex than that of the shear modulus μ, as it requires the introduction of different rheological models of the medium that consider frequency dependence of the SWV. Thus, it provides more information on tissue behavior than the simple mapping of the group velocity (μ = ρ × Vs^2^). Here, based on frequency dependence changes of the shear-wave speed, we observed higher dispersion (greater slope) of shear-wave speed with fatigue (post-exercise) than that at pre-exercise or post + 20’. The mean of the slope was 682.3 ± 646.5% higher immediately after exercise and 360.1 ± 383.7% higher at post + 20’ than baseline. These results are in accordance with those obtained by Deffieux et al. (2008), showing that SWS signal processing clearly depicts the viscous phantom as being highly dispersive, represented by a greater slope over the range of frequencies. Based on this relationship, we quantified the local resting VL η, which increased after fatigue (from ∼6.5 to ∼15 Pa × s) and returned to baseline 20 min after exercise. These results were also accompanied by a prolonged relaxation time and velocity (HRT and MRTR), calculated from the single evoked torque, suggesting that higher η may be related to reduced muscle fiber relaxation and a lower cross-bridge detachment rate, affecting muscle function. However, the HRT represents a global index of the muscle force of the entire quadriceps and not only that of the VL.

### Limitations and Perspectives

While this study provides scientific evidence of changes in VL μ and η with fatigue, it had methodological limitations: the entire quadriceps complex was not evaluated, limiting the interpretation of our results concerning other synergistic muscles (*rectus femoris* and *vastus medialis*), and the mathematical model used to estimate the elastic and viscous moduli assumes homogeneity and isotropy of the medium, whereas muscle is an anisotropic material. Anisotropy, assumed to be transverse isotropic in muscles, was not significant, as we investigated the imaging plane in the axis of the fibers. Moreover, no intermediate recovery measures were made, limiting interpretation about the time course of the viscoelastic parameters after the fatigue protocol representing restoration of the force transmission capacities. Finally, after the reproducibility tests, we found relatively high values for CV (>20%) mainly for VL μ and η. Generally, the criteria for CV is considered to be < 15% compared to the criteria for example for ultrasound studies (Van Hooren et al., 2020). This high level of CV is possibly due to ultrasound probe positioning which could have been moved during contractions despite the probe fixation system used or anisotropy of the muscle tissue. Moreover, in the present study, muscle viscosity was estimated after delivering four pushing beams by the ultrasonic probe at different levels of the region of interest as presented in Figure 2B. It is possible that the high CV values obtained were explained by the difference in the lateral distance (time of propagation) of each pushing beam affecting the analyzed velocity of the shear waves and its attenuation at different frequencies. However, VL η seems to be an interesting measure because it is sensitive to mechanical changes with fatigue despite the high level of CV (Figure 5; *P* < 0.01). For all participants, VL η values increased after the end of the fatigue protocol and decreased with the recovery (post + 20). A new SWS sequence needs to be developed in order to deliver several pushing beams in the left side of the ultrasound probe in order to ensure reproducibility of values.

Although these results must be interpreted with caution, they show promise in helping to gain a better understanding of the changes in the viscoelastic properties of muscle with fatigue. Moreover, studies focused on the mechanical properties of mechanical evoked responses coupled to B-mode ultrasound (ultra-fast sequences, very high frame rate) and their relationship to muscle stiffness and viscosity are required to gain insights about modifications in the force transmission capacity of individual muscles and fatigue.

## Conclusion

We have shown that VL muscle stiffness is strongly reduced by fatigue, both at rest and when active, reflecting higher muscle compliance. Moreover, such higher muscle compliance was accompanied by a greater dispersion of the shear waves, reflecting a more viscous muscle. *In vivo* evaluation of tissue viscosity may play a significant role in introducing frequency-dependent changes (dispersion) in muscle tissue, and parallel changes in elasticity may significantly alter the mechanical behavior of the tissue. The quantitative study of the viscoelastic properties of muscle may have important implications in sports medicine, as well as other fields of medicine, for better understanding the development of muscle fatigue and various pathological conditions, such as muscle dystrophy, motor neuron diseases, and inflammatory and metabolic myopathies.

## Data Availability Statement

The datasets generated from this study are available on request to the corresponding author.

## Ethics Statement

This study was approved by the scientific leadership of the French Armed Forces Biomedical Research Institute and the local Ethics Committee (CPP Ile de France VI). All experiments were conducted in accordance with the Helsinki Declaration Schuklenk, 2001.

## Author Contributions

EC, KC, JS, and SG-V designed the study, performed the analysis and interpretation of the data, and drafted the manuscript. AM, KC, CB, JS, and SG-V participated in the data collection. J-LG, LP, MO, AM, KC, and CB critically revised the manuscript.

## Conflict of Interest

J-LG is a scientific consultant for Supersonic Imagine.

The remaining authors declare that the research was conducted in the absence of any commercial or financial relationships that could be construed as a potential conflict of interest. The authors declare that the results are presented clearly, honestly, and without fabrication, falsification, or inappropriate data manipulation.
